# Step-by-step approach to reopening a Brazilian higher education institution during the COVID-19 pandemic

**DOI:** 10.1590/0034-7167-2021-0807

**Published:** 2022-06-24

**Authors:** Maria Clara Padoveze, Regina Szylit, Maria de Fátima Fernandes Vattimo, Erica Gomes Pereira, Lúcia Yasuko Izumi Nichiata

**Affiliations:** I Universidade de São Paulo. São Paulo, São Paulo, Brazil.

**Keywords:** COVID-19, Communicable Disease Control, Schools, Nursing, Pandemics, Health Planning, COVID-19, Control de Enfermedades Transmisibles, Facultades de Enfermería, Pandemias, Planificación en Salud, COVID-19, Controle de Doenças Transmissíveis, Escolas de Enfermagem, Pandemias, Planejamento em Saúde

## Abstract

**Objectives:**

to describe the experience of reopening a Brazilian higher education institution during the COVID-19 pandemic.

**Methods:**

experience report of a step-by-set approach to reopening a nursing higher education institution in São Paulo, Brazil, from May 2020 to May 2021.

**Results:**

the plan was created and operated by a group including students, professors, and technical-administrative workers. Weekly or by-weekly meetings occurred according to changes in the epidemiological situation and the needs to review the local technical and political agreements.

**Conclusions:**

we suggest that reopening plans during the COVID-19 pandemic should be politically and technically legitimated by all members of the community of a higher education institution so that they can take place quickly and sustainably. The early identification of COVID-19 cases and the adoption of local administrative measures are necessary to reduce the risk of outbreaks.

## RESUMO

**Objetivos:**

descrever a experiência de reabertura de uma instituição de ensino superior brasileira na pandemia de COVID-19.

**Métodos:**

relato das experiências vivenciadas no plano de abordagem passo a passo para reabertura de uma instituição de ensino superior brasileira de Enfermagem em São Paulo, Brasil, no período de maio/2020 a maio/2021.

**Resultados:**

o plano foi construído e operacionalizado por um grupo com estudantes, docentes e servidores técnico-administrativos. As reuniões semanais ou quinzenais ocorreram conforme as mudanças no panorama epidemiológico e a necessidade de revisão das ações técnicas e políticas locais.

**Conclusões:**

sugerimos que o plano de reabertura na pandemia de COVID-19 seja legitimado política e tecnicamente entre todos os membros da comunidade de uma instituição de ensino superior para que ocorra, de forma ágil e sustentada, a identificação precoce de casos de COVID-19 e a adoção de medidas administrativas locais visando reduzir o risco de surtos.

## RESUMEN

**Objetivos:**

describir la reapertura de una institución de enseñanza superior brasileña en la pandemia COVID-19.

**Métodos:**

informe de las experiencias vividas en el plan de abordaje paso a paso para reapertura de una institución brasileña de enseñanza superior de Enfermería en São Paulo, Brasil, de mayo/2020 a mayo/2021.

**Resultados:**

el plan fue construido y puesto en marcha por un grupo con estudiantes, profesores y servidores técnico-administrativos. Las reuniones semanales o quincenales se producían en función de los cambios en el panorama epidemiológico y de la necesidad de revisar los pactos técnicos y políticos locales.

**Conclusiones:**

sugerimos que el plan de reapertura se legitime política y técnicamente entre todos los miembros de la comunidad de una institución de enseñanza superior para que se produzca, de manera ágil y sostenida, la identificación precoz de casos y la adopción de medidas administrativas locales encaminadas a reducir el riesgo de brotes.

## INTRODUCTION

The COVID-19 was recognized as a pandemic by the World Health Organization (WHO) in March 11, 2020, leading to the urgent implementation of measures to halt the spread of the virus SARS-CoV-2 in teaching institutions of all levels^(1)^. As the epidemic advanced, non-pharmacological preventive measures, such as masks, social distancing, and hand hygiene were essential to consider reopening these institutions^(2)^.

International experiences showed that it is necessary to combine different strategies to reduce the dissemination of the virus and lead to massive adherence of practices to control and prevent the infection by SARS-CoV-2^(3)^. As a result, in-person education had to be adapted to the local, regional, and national health contexts, with the addition of technological resources for remote teaching. It is crucial to consider the obstacles that emerged during the closing of these institutions and in the process of reopening them^(4)^.

A document-based study in 19 countries of the European Union, which are part of the G20, reiterates that reopening educational institutions would be valid if protective directives are implemented. Without adequate protection, the risks of reopening can significantly increase the impact of COVID-19 on the population^(5)^.

Even in countries where universities continued to have on-site courses such as Taiwan, available guidance secured the safety of all university community members through means such as task forces carrying out risk triages, and searching for travel history, line of work, contacts, and groups. Other directives include health self-monitoring and quarantine if applicable; general hygiene measures (including the use of masks in indoor environments); principles of good ventilation and sanitization; reporting of suspected cases; political and technical consensus for closing, reopening, and established schedules for the replacement of missed classes when needed^(6)^.

Considering the gap of knowledge about the new disease, and the fact that non-pharmacological measures being adapted to the Brazilian context could only be effective in preventing infections if they were massively adopted by the population, higher nursing education institutions have faced several challenges. In addition, nursing students had to continue their professional training remotely while obeying the determinations of national health authorities in the three spheres of government (federal, state, and municipal)^(7)^.

The detailed description of local management experiences in a Brazilian Nursing higher education institution for sustainable reopening during the international health emergency of COVID-19 may be of interest for the prevention and management of future epidemics.

## OBJECTIVES

To describe the experience of reopening a Brazilian higher nursing education institution during the COVID-19 pandemic.

## METHODS

This article is an experience report on the step-by-step approach to reopening the School of Nursing at the University of São Paulo (SNUSP) in São Paulo, Brazil. As universities start attempting to reopen fully, this publication is justified due to its contribution to the knowledge about the essential bases of a safe return, dedicating close attention to the details inherent to theoretical, in-person, practical nursing teaching.

The SNUSP is one of the 42 units of the University of São Paulo, the largest university in Latin America. The School of Nursing has 80 years of history. It is divided into four departments, with approximately 615 students (327 in graduation, 288 in postgraduation), 64 professors (17 temporaries, 47 effective) 97 technical and administrative workers, and 24 outsourced workers (cleaning and security), in addition to 18 resident professionals and countless researchers who attend the institution.

The SNUSP, even before general university recommendations, instituted on March 11, 2020, the Work Group (WG) SNUSP COVID-19, aimed to gather information and operationalize the guidance of local sanitary authorities. The work of this group was helpful in the elaboration of the sanitary plan for the gradual resumption of in-person activities in the institution. On March 17, 2020, according with university directives, in-person teaching activities were suspended, as well as scientific events, public attendance, and any other event that would lead to agglomeration. This meant that in-person activities in all eight campi of the university, which are spread in 15 cities of the state of São Paulo, were stopped, impacting 89 thousand students, 5.8 thousand professors, and 14 thousand technical and administrative employees^(8)^.

The premise for the elaboration of a local plan was aligned with the São Paulo University plan; its later updates were based on the São Paulo Plan, from the Government of São Paulo^(9)^. The aim of the WG was to elaborate, execute, and follow up on the local sanitary plan whose objective was to structure the progressive resumption of academic and administrative in-person activities with as much safety as possible. The article refers to the period from May 2020 to May 2021, even though the sanitary plan is in full revision until the preparation of this report.

The authors of the article participated in the elaboration and development of the step-by-step plan to reopen the institution. The experience during the first year of implementation of this plan was analyzed in light of the documents available on the website of the institution, in September 2021.

## RESULTS

**Figure 1:**
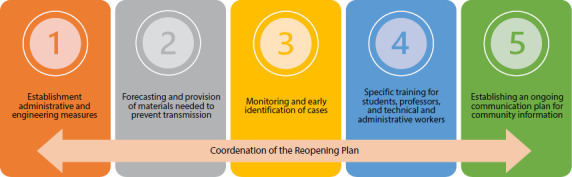
Scheme of the pillars of the Reopening Plan from the School of Nursing at the University of São Paulo, August, 2020

The WG was coordinated by professors from the Nursing School and was formed by professors, technical and administrative workers, graduation and post-graduation students, and post-doctoral researchers. This group met weekly or biweekly according to changes related to the epidemiological situation. As for its premise, the WG considered that the elaboration of the plan must be based on scientific knowledge and the regulations from sanitary authorities, meaning that it needed updates as new knowledge or recommendations became available. Five basic pillars were defined to elaborate the plan for a gradual resumption of in-person activities in the institution. These pillars were considered inextricably linked. They fulfill the purposes provided for in the bylaws concerning teaching, research, and the extension of university services to the community, as Figure 1 shows^(10)^. The members of the WG were distributed within the five pillars.

The full content of the plan can be accessed on the address https://sites.google.com/usp.br/gtee-covid-19.

### Pillar 1 - Establishing administrative and engineering measures

As one of the main elements to be included in the plan, the WG saw the need to adopt preventive measures in shared spaces, as international, national, and local health authorities indicated^(1-2,9,11)^.

Administrative and engineering measures to interrupt the transmission chain of the disease and decrease the risks in the dependencies of the SNUSP were taken. The physical spaces and work processes, such as classrooms and labs, were reorganized, with the acquisition of acrylic barriers, space signaling, the definition of general hygiene measures, and ventilation, cleaning, and cleansing measures to be adopted all sectors individually. Finally, guidance on telework and in-person classes (simultaneous or not) was given.

Two elements were adopted in the definition of proper measures: hierarchy of the controls of environment occupation risk (tec2) and estimates of potential risk in the activities carried out in the SNUSP (Chart 1).

**Figure 2:**
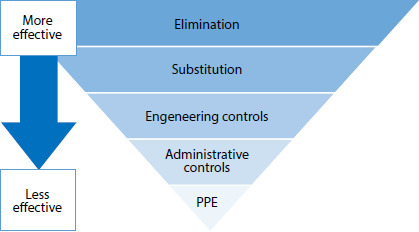
Hierarchy of environment occupation risk controls

**Table 1:** Potential risk in activities related to the School of Nursing at the University of São Paulo, August, 2020

Estimated level	Description
High	Refers to direct health care assistance during teaching, extension, or research, regardless of whether the patients being attended have a known or suspected COVID-19 diagnosis. Includes hospital assistance in Primary Care, home care, mental health, and other health services.
Medium	Refers to activities that require the gathering of people. Includes activities in teaching labs, classrooms, attention to the public, and cleaning.
Low	Refers to activities developed individually or in reduced spaces (1 person per 7 m^2^).

Based on these elements measures were established to isolate people from risk sources or changes in processes to minimize these risks. They included signs on the spaces indicating a minimum distance of 1.5m to avoid agglomeration, alcohol gel dispensers for hand hygiene, blockage in collective drinking fountains, thermometer purchases, reinforcement of environmental cleaning, in addition to other measures of environmental organization.

Furthermore, administrative measures were found to aid in reducing the stress-related to work activities when properly planned, including the participation of all those involved in decision making. As a result, actions were taken to support the management of stress, fatigue, and anxiety, such as creating support groups in the institution itself or facilitating access to support groups in other institutions.

### Pillar 2 - Forecasting and provision of materials needed to prevent transmission

The WG, as it instituted the pillar of prevision and provision of materials to prevent transmission, carried out an analysis about the need for acquiring and providing personal protection equipment (PPE) to undergraduation students, professors, and technical and administrative workers.

The university provided a kit with a fabric mask and a face shield. In addition, the pillar organized spreadsheets forecasting the need to acquire N95 masks for the students and internship supervisors who could be exposed to aerosol generating procedures, taking into consideration the number of individuals, the number of courses, and the duration of the activities (number of days). Organizational supply mechanisms were quickly established, including a flowchart for the request and provision of PPE, a PPE control and delivery form, and recommendations for the use, cleaning, disposal, and storage of PPEs.

### Pillar 3 – Monitoring and early identification of cases

The WG created the monitoring plan of the SNUSP community, including professors, students, technical and administrative workers, and outsourced professionals who work in the school. This was done using a passive method consisting of community members awareness regarding the most frequent symptoms to monitor flu-like illness suspected cases^(10)^. Additionally, an active component to measure temperature was operationalized at the institution’s entrance. The main goal of the monitoring was the early identification of cases to avoid the dissemination of the virus in the SNUSP spaces and reduce the risk of COVID-19 outbreaks in the institution.

Periodically, community members received messages to encourage passive monitoring through institutional emails and information bulletins. Directives recommended daily filling of a Google Form® with questions about signs and symptoms and the presence of confirmed COVID-19 cases among shared households. The WG agreed upon the algorithm to screen for issues, and the specific actions to be carried out for each respondent were managed daily, via telephone, from Monday to Friday.

The isolation recommendations (for suspected or confirmed cases) or quarantine (household contacts with suspected or confirmed cases) were individually provided for the respondents, who were advised to inform their leaders or the supervisor/ coordinator of courses. In addition, during the phone contact with the respondents, they were advised about the need to be absent from in-person activities in the institution and to seek clinical evaluation in a health services. Periodically, local data were analyzed to ensure that the measures in place effectively prevented COVID-19 dissemination, focusing on the prevention of outbreaks in the institution.

### Pillar 4 - Specific training for students, professors, and technical and administrative workers

The WG recommended the organization of training sessions about “Measures to protect against COVID-19 in the gradual resumption of in-person SNUSP activities”, preceding the progressive return of teaching and university extension activities. All professors, technical and administrative workers, and outsourced workers post-doctoral researchers, graduation, and post-graduation students had to undergo these training sessions. In the coordination of the WG, the training was developed in the Google Classroom® platform through asynchronous distance teaching, using slides, videos recorded in the institution, and other recommended videos. In addition, participants sent their home made videos about the topics discussed in the course, and there were multiple-choice questions and lab practices.

The general objective of these training sessions was to provide information and encouragement to the construction of reflection and the implementation of essential content to prevent and control infection in different contexts, applied to the NS in the prevention of COVID-19. Therefore, four modules were defined about the content about respiratory syndromes, focusing on COVID-19: 1) New coronavirus and COVID-19; 2) Essential care; 3) Application in different internal SNUSP environments; 4) Application in different external contexts.

The WG followed up the adherence to of the NSUSP community members to the trainings and developing actions to improve it, such as encouraging mechanisms (messages to encourage and congratulate participants for finishing modules, participant certificates) and check in regarding whether the supervisors of the internship of the students and courses coordinators were participating in the modules.

### Pillar 5 - Establishing an ongoing communication plan for community information

The WG considered that the communication is an essential pillar of the plan, thus established actions for a better dialogue between the WG and the SNUSP community members. The objectives of the plan were: to ensure that information about the development of the “Plan for the Resumption of the Academic and Administrative Activities” was known by the SNUSP community and its potential visitors; allowing the appreciation of the actions proposed by the “Plan for the Resumption of the Academic and Administrative Activities”; increasing awareness about preventive measures; mediating conflict resolution; and facilitating communication, collaboration, and teamwork.

The management of official communication with the SNUSP community and its external community was carried out using the technological resources available in the platform Google USP — G Suite for Education. To disseminate knowledge and open a broad debate about the plan, the WG created an institutional email; elaborated educational and informative materials available in the form of bulletins, infographics, banners, and others; carried out weekly or biweekly meetings using the Google Meeting platform with the NSUSP community; and created, with the participation of WG members and post-graduation students, materials for digital platforms called “On Duty for Coronavirus - Information Pills” and the “*Podcast EE: Notas da Pandemia*” (NS Podcast: Pandemic Notes). Finally, the WG recommended developed an open website aiming at to build repository of documents and actions and as one of the means of communication between WG and SNUSP community.

## REFLECTING ON THE EXPERIENCE: THE LESSONS LEARNED

The experience in the operationalization of the plan showed that resuming in-person activities requires thorough planning, and the creation of a time-efficient local group to guide the initiative, who must work fast to break infection transmission chains in the institution. In addition, the results showed that the engagement of the entire community was paramount for the plan to be successful while respecting international findings^(6,11,12)^.

Two years have passed since the beginning of the pandemic emergency, which is still taking place. We have learned how necessary it is for technical responses to be fast and for communication to be fast enough to deal with daily problems. Therefore, it is important to highlight the need for agile mechanisms for conducting the WG, as each member plays their role and has a well-defined scope of action, as shown by successful international experiences in teaching institutions that resumed their activities during the pandemic^(5,7)^.

Considering all misinformation and fake news, teaching institutions keep the scientific evidence as a guide when making decisions to reduce outbreaks and mitigate the spread of the virus. The plan’s development showed that it can only be brought into effect if articulated and considering the limitations inherent to managing large-sized institutions.

## CONCLUSIONS

The experience of reopening a Brazilian higher nursing education institution during the COVID-19 pandemic is still challenging for the legacy of the local governance planning, prevention and management of future epidemics. Therefore, we suggest that reopening plans during the COVID-19 should be politically and technically legitimated by all the members in the community of a higher education institution, so it can be fast and sustainable, with early identification of cases and the adoption of local administrative measures to reduce the risk of outbreaks.
